# Agreement between official race data and a high-end sports tracker for a Paralympic rower

**DOI:** 10.1038/s41598-026-45846-x

**Published:** 2026-04-11

**Authors:** Fredrik Mentzoni, Thomas Losnegard

**Affiliations:** 1 The Norwegian Olympic Sports Center, The Norwegian Olympic and Paralympic Committe and Confederation of Sports, Oslo, Norway; 2 Department of Physical Performance, Norwegian School of Sports Sciences, Oslo, Norway

**Keywords:** Race analysis, Performance analysis, GNSS, IMU, Measurement uncertainty, Tracking, Engineering, Mathematics and computing

## Abstract

Race data containing speed and stroke rate per 50m, provided by World Rowing, were compared with measurements using an ASI AdMos sensor, which is a high-end sports tracker, to assess the agreement between the two measurement systems. Twelve races in 2022, 2023, and 2024 were considered. All measurements were of the same rower competing in the Paralympic PR1 W1x class. The race data speed was compared with speed calculated from the GNSS data of the AdMos sensor, while stroke rate was compared with the stroke rate calculated from the surge acceleration of the IMU data of the AdMos sensor. Comparisons were made for each 50m segment. Additionally, official 500m split times were used to assess the accuracy of both the race data and the AdMos measurements. The absolute difference between the race data and the AdMos measurements was within rounding tolerance in 312 of 480 segments (65%) for speed and 410 of 480 segments (85%) for stroke rate. The comparison of mean speed per 50m segment revealed a mean absolute difference (MAD) of 0.05ms$^{-1}$ (mean absolute relative difference: 1.6%). The speed difference was largest in the first 50m of the race, with a MAD equal to 0.39ms$^{-1}$ (12%). If comparing the race data with the AdMos speed of the last stroke before each segment end, the difference in the first 50m reduced to 0.12ms$^{-1}$ (3.3%). The MAD in stroke rate was 0.3 strokes per minute (1.0%). Compared to the official 500m split times, the MAD of the AdMos was 0.3s (0.2%), whereas calculation of the 500m splits from the race data yielded a MAD equal to 1.0s (0.7%). World Rowing’s official 50m race data showed good agreement with the high-end sports tracker in most segments, but the systems diverged considerably for speed in the first segment. Practitioners should verify data characteristics before using official race data for high-precision biomechanical or pacing analyses.

## Introduction

In rowing regattas organized by the World Rowing federation, race data are commonly made available post-race. The race data contain speed and stroke rate for each rower of the regatta, with each quantity given every 50m. Nolte^1^, explains that the provided race data represent “the average of all 50 m segments” during the 2000 m race, understood as the arithmetic mean of the speed and stroke rate of each 50 m segment, starting with 0 to 50 m, then 50 m to 100 m, and so on for all 40 segments ending with 1950 m to 2000m. The detailed segmentation provides a valuable tool for post-race analysis. It allows rowing crews to evaluate their performance and compare it with competitors. One example is the assessment of pacing, i.e., the distribution of power during the race, which is closely linked to the distribution of speed^1^ and stroke rate^2^. Several studies have assessed pacing in rowing, but typically only 500 m split times are analyzed^3–8^. However, as argued by Nolte^1^, pp. 252–253, assessing the speed every 50 m may reveal pacing variations that are not present in the 500 m splits. Furthermore, the race data include the stroke rate, which is an important aspect of rowing performance^2^. When both speed and stroke rate are available for each 50 m segment, the distance per stroke can be estimated throughout the race. This parameter is used by coaches and sport scientists to assess rowing efficiency and technique^9^. Importantly, the value of the race data depends on the expected measurement uncertainty; however, to the authors’ knowledge, no published studies have evaluated these data against other high-end measurement systems.

**Fig. 1:**
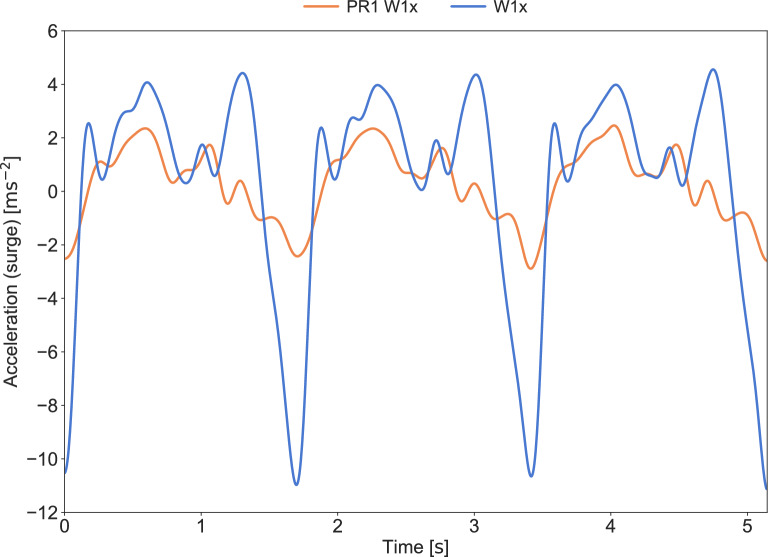
Comparison of filtered surge acceleration between an Olympic class W1x rower and a Paralympic class PR1 W1x rower, measured using an ASI AdMos sensor. Data is processed with a 4th order Butterworth low-pass filter, cut-off frequency 6 Hz, cf^10^.. Both rowers are at 35 strokes per minute during a 2000 m regatta. Note the difference in local minima, associated with the catch of the stroke, at time equal to 0, 1.7 s, 3.4 s, and 5.1 s.

The official race data provided by World Rowing appear to be collected via portable, boat-mounted units that are attached to each vessel prior to competition^11^. While the internal processing of these units is proprietary, the 50 m speed data are reported to be based on global navigation satellite system (GNSS) receivers^1^. A method to estimate the mean stroke rate at 50 m intervals is to utilize inertial measurement units (IMU). However, as the exact specifications and algorithms used for the official World Rowing datasets are not publicly disclosed, they remain a “black box” for practitioners. This lack of transparency necessitates independent verification to ensure the data’s reliability for performance analysis.

The stroke rate can be determined by analyzing the surge acceleration of the boat, identifying the start of each stroke as a local minimum in the acceleration associated with the catch, that is, the start phase of the stroke when the rower puts the blade(s) in the water^10,12^. The minimum acceleration during a stroke can be substantial, and is generally easy to identify, e.g., by using peak detection (data analysis)^13^. Notably, in Paralympic fixed-seat rowing, the peak in minimum acceleration associated with the catch is typically less pronounced. This could make it more challenging to determine the stroke rate. To illustrate, a comparison of the boat surge acceleration of a Paralympic class single sculler (PR1 W1x) and an Olympic class single sculler (W1x) is presented in Fig. 1.

The present study aims to evaluate the agreement between the official race data provided by World Rowing and a high-end sports tracker previously evaluated for use in comparable outdoor sports^14^. We assess the agreement by comparing both the speed and stroke rate of the official race data with concurrent high-resolution measurements using the ASI AdMos sports tracker. We hypothesized that the two systems would provide similar outputs for both speed and stroke rate per 50 m throughout the 2000 m race, although we anticipated potentially greater discrepancies in the estimated stroke rate than in speed given that the rower used a fixed seat. Ultimately, our assessment aims to determine both the expected uncertainty and the practical usefulness of the official speed and stroke rate data, providing athletes, coaches, and practitioners with a clearer understanding of the race data’s validity for performance evaluation and post-race analysis.

## Methods

The present study evaluated the agreement of openly available race data with data collected using an ASI AdMos sports tracker. The study can be considered a simple case study, as only one rower, i.e., “participant”, was included. Because the race data (speed and stroke rate) are not considered sensitive personal health information, formal ethical approval from an institutional or governmental review board was not required for this specific analysis. Nevertheless, the study was conducted in accordance with the Declaration of Helsinki. The participant provided written informed consent for the use of their data and was notified that they could withdraw this consent at any time.

Twelve races, presented in Table 1, from six different rowing events between August 2022 and April 2024, were considered. These events comprise all international competitions organized by World Rowing in which the athlete participated during the data collection period (August 2022 – April 2024). In each of the six events, one heat or preliminary and one final were analyzed. In each race, an ASI AdMos sensor was installed in the boat by a member of the rower’s support team, following a standardized protocol to ensure consistent data collection throughout the study period. The sensor was configured to record GNSS data (position) at a frequency of 10 Hz and IMU data (accelerations and rotations) at 200 Hz. This sensor has been evaluated in alpine skiing and is expected to perform well in open-area sports suitable for reliable GNSS measurements, such as rowing^14^. Notably, in alpine skiing, it showed low instantaneous speed errors (median $< {0.04}{\hbox {ms}^{-1}}$), supporting its suitability for accurate speed tracking in comparable environments. Further details on the sensor, including its applicability in sports, is presented by Jølstad et al.^14^. The AdMos data were post-processed and analyzed using Python 3.11. Official 50 m race data, 500 m splits and 2000 m times were retrieved from the World Rowing website^15^. In addition to the comparison between the 50 m race data and the AdMos sensor, the official 500 m split times were compared with the estimated split times from the 50 m race data and the AdMos measurements. The official race data are available in the documents sections for each event, cf. Table 1, on the World Rowing website^15^.

**Table 1:** Overview of the twelve races analyzed in the study, spanning six rowing events from August 2022 to April 2024. For each event, both a heat (or preliminary) and a final race were included.

Race label	Date	Event	Time
E22p	2022-08-11	European Rowing Championships	11:50.83
E22f	2022-08-13	European Rowing Championships	10:59.89
W22h	2022-09-20	World Rowing Championships	10:16.28
W22f	2022-09-25	World Rowing Championships	10:07.58
E23h	2023-05-25	European Rowing Championships	10:17.04
E23f	2023-05-27	European Rowing Championships	10:05.80
WC232h	2023-06-16	World Rowing Cup II	10:09.92
WC232f	2023-06-18	World Rowing Cup II	09:47.83
W23h	2023-09-05	World Rowing Championships	09:54.08
W23f	2023-09-10	World Rowing Championships	10:05.91
E24p	2024-04-25	European Rowing Championships	10:00:39
E24f	2024-04-27	European Rowing Championships	10:52:40

The GNSS measurements were used to calculate the time spent on each 50 m (and 500 m) segment. The distance rowed was calculated from the 10 Hz records of latitude and longitude. The speed of the boat was calculated from distance and time using a 2nd order accurate central difference scheme. Based on trial and error from previous measurements of the considered rower, the start of the race was set to 0.7s prior to the boat reaching a speed of 1ms$^{-1}$. The finish was determined as a 2000 m straight line from the coordinates at the start. Each 50 m (and 500 m) segment was defined in a similar way, e.g., the segment from 1350 m to 1400 m was set to start at the coordinates that were 1350 m away from the set start coordinates, and the end of the segment was at the coordinates that were 1400 m away from the set start coordinates.

The measured boat acceleration in surge was used to determine the stroke rate. A 4th order Butterworth low-pass filter with a cut-off frequency of 6 Hz was applied to the raw measurements, similar to the method described by Holt et al.^10^. Peak detection was performed on the negative filtered surge acceleration to identify the peaks corresponding to the catch phase of each stroke. Constraints were set on the minimum time between two consecutive peaks (1.2s) and the minimum amplitude (1.0ms$^{-2}$) to ensure the accuracy of the peak identification. The first constraint effectively limits the stroke rate to no more than 50 strokes per minute, while the latter ensures that only negative minima with more than 1.0ms$^{-2}$ in absolute value are considered peaks associated with the catch, cf. Fig 1.

The pacing variability, quantified as the coefficient of variation ($\textrm{CV}$)^7^, was assessed. In each race, the pacing variability was calculated using the five different sources of data, that is, mean speed and stroke rate per 50 m segment from both race data and AdMos measurements, as well as the official 500 m split times. The correlation coefficient (*r*) between the pacing variability obtained from the different data sources was calculated based on the $\textrm{CV}$ in the twelve races. The purpose of this assessment was to evaluate the consistency of the pacing variability obtained from different data sources. To further assess the agreement between the two systems, Bland–Altman analyses were performed for the speed (race data vs. sports tracker) and stroke rate (race data vs. sports tracker) $\textrm{CV}$. For these analyses, the mean bias and 95% limits of agreement (LoA) were calculated. The LoA were determined using the critical value of the t-distribution with $n-1$ degrees of freedom ($n=12$ races; $t = 2.201$). The same procedure was applied when assessing the agreement for the 480 individual 50 m segments; however, due to the larger sample size, the critical value for these segments ($t = 1.965$) was nearly identical to the standard z-score.

In addition to comparing the mean stroke rate and mean speed for each 50 m segment, the mean speed and stroke rate of the last stroke before the end of each segment were compared with the race data. The purpose of this assessment was to compare the race data with AdMos measurements taken closer to the segment end.

Note that the stroke rate in the race data is reported as a whole number of strokes per minute (spm), without decimals. Therefore, any absolute difference smaller than 0.5 spm can be considered equivalent within rounding tolerance. For example, if the race data reports a stroke rate of 31 spm, a stroke rate calculated from the AdMos measurements is considered equivalent within rounding tolerance if it falls within the interval [30.5, 31.5) spm. Similarly, speed is reported in meters per second with one decimal place in the race data, meaning that any absolute difference smaller than 0.05ms$^{-1}$ is considered equivalent within rounding tolerance.

## Results

A summary of the differences in speed and stroke rate between the race data and the AdMos measurements are provided in Table 2. The assessment is based on the 480 calculated differences (12 races times 40 segments in each race).

**Table 2:** Summary of the 480 differences in speed and stroke rate between the race data (RD) and the AdMos (AM) measurements, i.e., RD - AM. The mean of the differences, the mean of the absolute value of the differences (MAD), the standard deviation of the differences (SD), the first (Q1; 25th percentile), second (Q2; median), and third (Q3; 75th percentile) quartiles,and the lower and upper limits of the 95% limits of agreement (LoA) are presented.

	Mean	MAD	SD	Q1	Q2	Q3	95% LoA
Speed [ms$^{-1}$]	0.013	0.050	0.083	−0.030	0.010	0.044	(−0.15, 0.18)
Stroke rate [spm]	0.02	0.29	0.36	−0.25	0.00	0.29	(−0.68, 0.72)

**Fig. 2:**
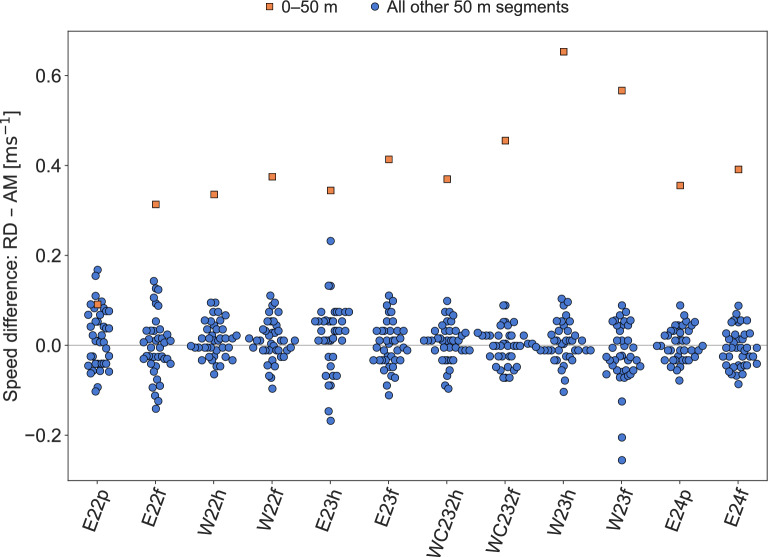
Difference in speed between the race data (RD) and AdMos (AM) measurements per race, i.e., $\text {RD} - \text {AM}$. Blue circular markers are used for all segments except the first which is presented using orange square markers. Notably, in all races except for E22p, the speed in the first 50 m segment is overestimated by more than 0.3ms$^{-1}$ in race data compared to the AdMos measurements.

**Fig. 3:**
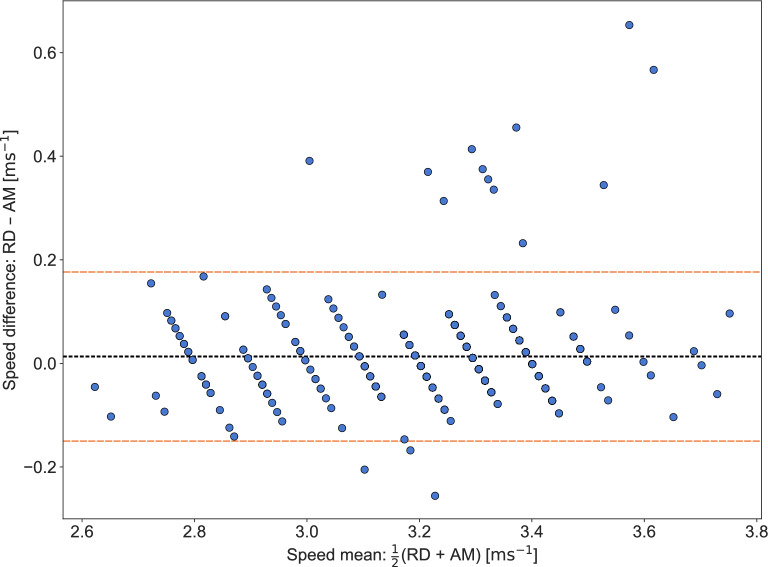
Bland–Altman plot of differences in speed between the race data (RD) and AdMos (AM), i.e., $\text {RD} - \text {AM}$. The bias, illustrated with a black dotted line, was 0.013ms$^{-1}$ and the lower and upper limits of the 95% limits of agreement (LoA), illustrated with orange dashed lines, were (−0.15ms$^{-1}$, 0.18ms$^{-1}$).

The mean absolute difference (MAD) in speed per 50 m segment was 0.050ms$^{-1}$, equivalent to a relative difference of 1.6%. The absolute difference fell within the rounding tolerance in 65% ($\frac{312}{480}$) of the segments. Notably, the first 50 m segment had the largest average speed difference, with a MAD of 0.39ms$^{-1}$ (12.4%). In 11 of the 12 considered races, the difference in speed was largest in the first 50 m segment. This is illustrated Fig. 2, in which differences in speed per race for each 50 m segment are presented, using orange square markers for the first segment (0-50m) and blue circular markers for all other segments. A Bland–Altman plot of the differences in speed between the official race data and the AdMos sports tracker is provided in Fig. 3.

When comparing the race data with the AdMos measurements of the last stroke before the 50 m mark, the MAD for the first segment decreased to 0.12ms$^{-1}$ (3.3%). However, applying this method across all segments resulted in an increased MAD of 0.069ms$^{-1}$ (2.1%). Additionally, this approach resulted in only 43% ($\frac{204}{480}$) of the segments being within the rounding tolerance of less than 0.05ms$^{-1}$.

**Fig. 4:**
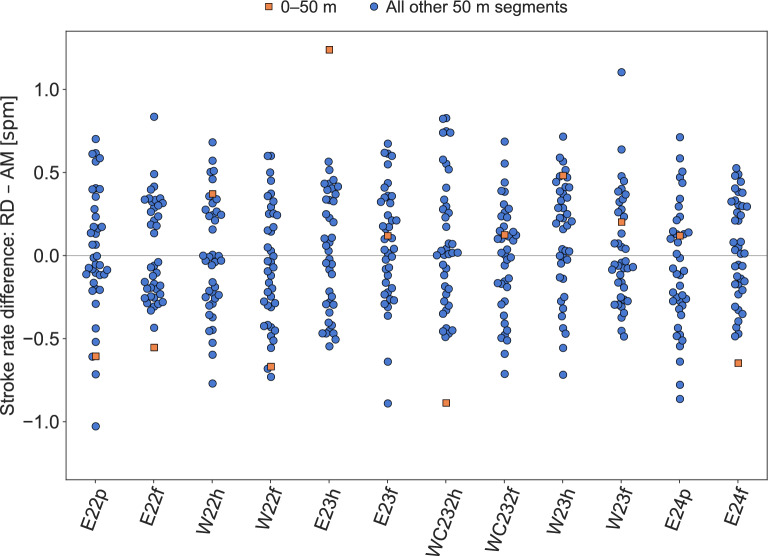
Difference in stroke rate between the race data (RD) and AdMos (AM) measurements per race, i.e., $\text {RD} - \text {AM}$. Blue circular markers are used for all segments except the first which is presented using orange square markers. The figure illustrate that the difference in stroke rate between the race data and the AdMos measurements is relatively evenly distributed among both races and segments, which was not the case for the speed.

**Fig. 5:**
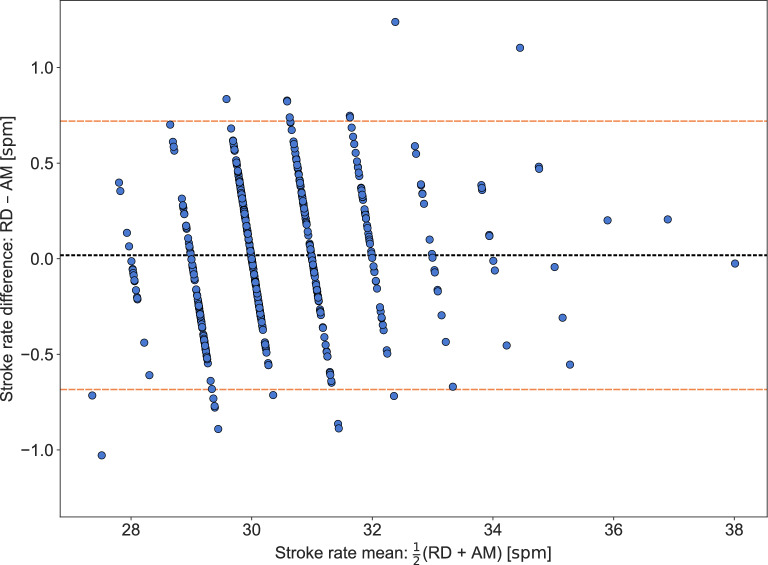
Bland–Altman plot of differences in stroke rate between the race data (RD) and AdMos (AM), i.e., $\text {RD} - \text {AM}$. The bias, illustrated with a black dotted line, was 0.02spm and the lower and upper limits of the 95% limits of agreement (LoA), illustrated with orange dashed lines, were (−0.68spm, 0.72spm).

The MAD in stroke rate between the race data and the AdMos measurements for the 480 segments was 0.29spm (0.96%). The absolute difference was within the rounding tolerance, that is, less than 0.5spm, in 85% ($\frac{410}{480}$) of the segments. Differences in stroke rate per race and per segment are visualized in Fig. 4, using the same color and marker coding as for the speed in Fig. 2. Additionally, a Bland–Altman plot of the differences in stroke rate between the official race data and the AdMos sports tracker is provided in Fig. 5.

The largest absolute stroke rate difference occurred in the first 50 m segment, with a MAD of 0.50spm (1.5%). Comparing the race data with the AdMos-measured stroke rate of the last stroke before the 50 m mark reduced the MAD for the first segment to 0.47spm (1.4%). However, applying this method across all segments resulted in an increased MAD of 0.42spm (1.4%). Furthermore, this approach yielded 66% $\frac{319}{480}$ of the segments having an absolute difference less than 0.5spm.

**Fig. 6:**
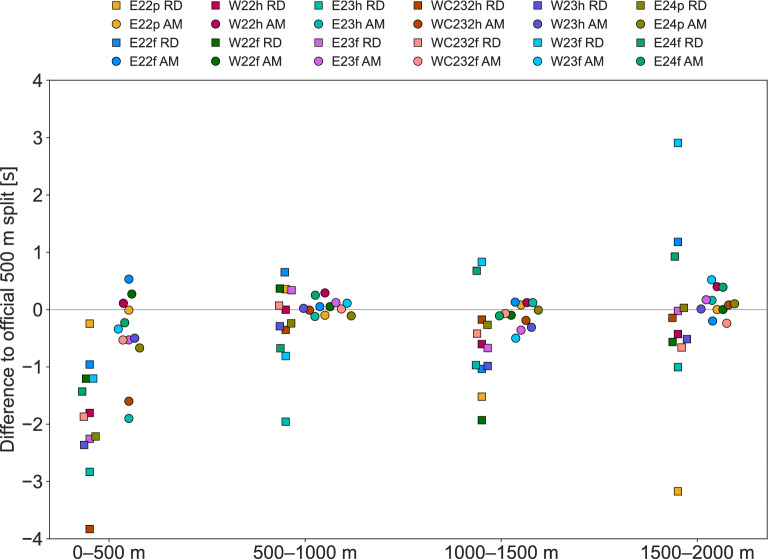
The difference to the official 500 m split time per 500 m segment for split times estimated based on the speed in the race data (RD; rectangles) and from the measured position with the AdMos sensor (AM; circles). Race labels are given in Table 1.

The differences to the official 500 m splits of the race data and AdMos measurements are presented in Fig. 6. Compared to the official 500 m split times, the MAD of the AdMos measurements was 0.27s (0.17%), whereas calculation of the 500 m splits from the race data speed yielded a MAD equal to 1.0s (0.67%).

The mean difference in 2000 m time between the AdMos measurements and the official race time was −0.39s; the MAD was 0.71 s (0.12%). The mean difference in 2000 m time between the race data and the official race time was −2.78s; the MAD was 3.06s (0.50%) That is, both the AdMos measurements and the race data predicted, on average, faster 2000 m time than the official timing.

The pacing variability ($\textrm{CV}$) was calculated based on the various data sources across the twelve races. Before performing the correlation and agreement analyses, the normality of all $\textrm{CV}$ distributions was confirmed using Shapiro–Wilk tests ($p \ge 0.05$ for all variables). The correlation coefficients between the $\textrm{CV}$ of the 500 m splits and the other data sources were $r = 0.81$ (race data speed), $r=0.56$ (race data stroke rate), $r = 0.89$ (AdMos speed), and $r=0.66$ (AdMos stroke rate).

**Fig. 7:**
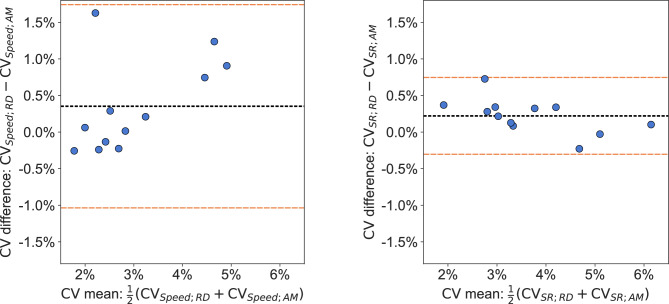
Bland–Altman plots for the pacing variability ($\textrm{CV}$) in the 12 races calculated based on speed (left) and stroke rate (right). In both plots, the difference is given as the $\textrm{CV}$ based on the 50 m race data (RD) minus the $\textrm{CV}$ based on the corresponding measurements per 50 m using the AdMos (AM) sports tracker. For $\textrm{CV}$ based on speed, the bias was 0.35% and the lower and upper limits of the 95% limits of agreement (LoA) were (−1.04%, 1.74%). For $\textrm{CV}$ based on stroke rate, the bias was 0.22% and the lower and upper limits of the 95% limits of agreement (LoA) were (−0.30%, 0.75%).

To evaluate the agreement in calculated pacing variability between the official race data and the AdMos sports tracker, Bland–Altman analyses were performed for the metrics measuring identical parameters at the same sampling resolution. These are illustrated in Fig. 7. The correlation between the pacing variability calculated from the race data speed and the AdMos speed was $r=0.88$, and the corresponding Bland–Altman analysis revealed a bias of ${0.35}{\%}$ with ${95}{\%}$ limits of agreement (LoA) from ${-1.04}{\%}$ to ${1.74}{\%}$. For stroke rate, the correlation in pacing variability calculated from the race data and AdMos was $r=0.99$, with the Bland–Altman analysis showing a bias of ${0.22}{\%}$ and LoA between ${-0.30}{\%}$ and ${0.75}{\%}$.

## Discussion

Official race data by World Rowing were compared to measurements using the ASI AdMos sports tracker to evaluate the agreement between the two systems. In total, 480 segments of 50 m were analyzed. In most segments (65% for speed and 85% for stroke rate), the absolute difference between the race data and the sports tracker was within the rounding tolerance. However, the agreement for speed was limited in certain segments, suggesting that the official race data and the sports tracker measurements do not consistently yield identical results for 50 m segments. An important finding was that the official race data consistently reported higher speeds in the first segment of the race, on average 12% higher compared to the sports tracker. In contrast, the race data stroke rate per 50 m corresponded, in general, to the mean stroke rate in each 50 m segment when assessing the filtered surge acceleration of the sports tracker. This was a particularly noteworthy finding, as the considered rower used a fixed seat, which potentially could have made it more challenging to identify the stroke rate from the boat surge acceleration. Contrary to our hypothesis, the agreement for stroke rate was remarkably high despite the fixed seat, while the largest discrepancies were observed in the speed data, in particular during the first race segment.

It is unclear how the speed and stroke rate were calculated in the race data by World Rowing. The lack of agreement between the race data and the sports tracker may suggest that the race data did not represent “the average of all 50 m segments”^1^, as is the common belief within the rowing community^1,9,16^. This was particularly evident when assessing the first segment, 0-50m, where the boat accelerates from rest to a speed close to the mean race speed. In all considered regattas, the race data suggested a higher mean speed for this segment as compared to the sports tracker. A possible reason for the disagreement in the first 50 m segment could be related the how the race data timing is initiated. The start of the timing ($t_0$) might be triggered when the boat reaches a certain speed, rather than being correctly synchronized with the actual start of the race. In addition to such synchronization errors ($t_0$), other technical factors may contribute to the observed discrepancies, such as GNSS signal acquisition lag during the rapid transition from a static to a dynamic state, or the influence of proprietary smoothing algorithms used in the “black box” official units. In such cases, when the boat crosses the 50 m line, the timing may not reflect the full duration required to cover the distance, which can lead to an overestimation of the speed for this segment. Note that in subsequent segments, speed changes from stroke to stroke are more subtle, and the differences between the mean speed of the segment and each stroke within the segment are generally smaller, especially if the rower maintains a relatively even pace. This may be another explanation why the differences between the race data and the mean speed calculated with the sports tracker were, in general, smaller in segments after the first 50m. Nevertheless, even if the first segment is ignored from the analysis, there are still 156 segments (${33}{\%}$) with an absolute speed difference to the sports tracker of more than the rounding tolerance.

The exact method used to calculate the speed and stroke rate in the race data remains unclear. Given the considerable speed changes of a rowing boat within each stroke^17^, it seems very unlikely that the instantaneous speed (i.e., speed measured during a very short time period, $\Delta t \ll {1}{s}$), as the boat crosses the end of a 50 m segment, is used. A more plausible hypothesis is that the speed in the race data represents the mean speed of the last stroke before the segment end. However, our analysis does not support this, as the level of agreement between the two systems decreased when comparisons were based on the last stroke before the segment end; the mean absolute difference increased from 1.6% to 2.1%, and the proportion of segments within the rounding tolerance decreased from 65% to 43%.

The additional time spent on the first 50 m, due to the acceleration from rest, is illustrated by Thompson^18^, Fig. 11.1, using 50 m split data from the World Championships of 2011. Depending on the boat class, the increase in time is typically from 1 s to 4 s compared to the mean 50 m split time^7,18^, resulting in a somewhat slower segment speed than the mean race speed. Moreover, Chu et al.^16^ shared 50 m race data from the World Championships of 2010–2017^19^. In that dataset, the reported speed in the first 50 m segment was typically slower than the mean race speed in the years 2010–2016, but this pattern changed in 2017. Official race data for 2018^20^ and onwards^15^ illustrate a similar pattern, that is, the reported speed in the first 50 m segment is no longer slower than the mean race speed. Consequently, it is plausible to infer that while the 50 m race data may previously have been based on the arithmetic mean of each segment, a different calculation method or measurement technique has likely been applied since 2017. This shift in the data’s characteristics further supports our finding of limited agreement between the official race data and the sports tracker in the first 50 m segment.

The agreement between the sports tracker and the official timing in 500 m splits and 2000 m time was, in general, high given the expected measurement uncertainty. The absolute difference was less than 1 s in all but two 500 m splits (96%). Both cases with higher discrepancies occurred in the first segment, from 0 m to 500m. One explanation for this can be the algorithm applied to setting the start time in the assessment of the GNSS measurements. However, this effect is unlikely the sole explanation for a 1.9s difference, as was found in one of the races. Another candidate is the alignment of boats at the start of the race. In race videos published by World Rowing^15^, it is evident that the boats are not always aligned at the appropriate start line. An example is the 2023 World Rowing Cup II, in which a different start line was used compared to when the same course hosted the 2021 European Rowing Championships, suggesting that the course distance in PR1 W1x races is not always equal to exactly 2000m.

The pacing variability in the twelve races was positively correlated between the various data sources. The variance in the CV of the 500 m splits explained 78% of the variance in the CV of the 50 m mean speed measured with the AdMos sensor. Surprisingly, the CV of the 50 m AdMos speed was better correlated with the CV of the 500 m splits than with the CV of the 50 m race data speed. The Bland–Altman analyses illustrate that pacing analyses based on speed may yield somewhat different results depending on whether official race data or the sports tracker is used, whereas analyses based on stroke rate show smaller discrepancies between the data sources. This is consistent with the separate assessments of speed and stroke rate performed in this study. Note the limitations of the pacing variability analysis as only 12 races and one rower were considered. However, our analysis suggests that the analysis of 500 m splits, despite their lower resolution compared to 50 m segment data, still provides considerable insights into pacing variability. The recommendation to assess shorter segments for detailed pacing analyses remains valid^1,9,16^; however, given the observed lack of agreement between the official 50 m race data and the sports tracker, one must be careful when interpreting these data. Consequently, coaches and sport scientists should verify data characteristics before using them for detailed biomechanical or pacing analyses.

## Conclusion

The official 50 m race data provided by World Rowing showed high agreement with the ASI AdMos sports tracker in the majority of analyzed segments. However, the two measurement systems did not consistently yield identical results across all segments. The agreement was particularly poor for the speed in the first segment of the race. The methods used by World Rowing to calculate speed and stroke rate remain unclear. Consequently, practitioners relying on the race data for post-race analyses should be aware of possible substantial discrepancies between different measurement systems. A key practical implication of these findings is that data characteristics should be verified before using official datasets for detailed biomechanical or pacing analyses.

## Data Availability

The official race data are available in the documents sections for each event on the World Rowing website. The corresponding measurements of 50 m speed and stroke rate using the AdMos sports tracker is available from the first author upon reasonable request.
